# The Effectiveness of a Web-Based Personalized Feedback and Social Norms Alcohol Intervention on United Kingdom University Students: Randomized Controlled Trial

**DOI:** 10.2196/jmir.2581

**Published:** 2013-07-24

**Authors:** Bridgette M Bewick, Robert M West, Michael Barkham, Brendan Mulhern, Robert Marlow, Gemma Traviss, Andrew J Hill

**Affiliations:** ^1^Academic Unit of Psychiatry and Behavioural Sciences, Leeds Institute of Health SciencesSchool of MedicineUniversity of LeedsLeedsUnited Kingdom; ^2^Division of BiostatisticsUniversity of LeedsLeedsUnited Kingdom; ^3^Centre for Psychological Services ResearchUniversity of SheffieldSheffieldUnited Kingdom; ^4^School of Health and Related ResearchUniversity of SheffieldSheffieldUnited Kingdom; ^5^School of ComputingUniversity of LeedsLeedsUnited Kingdom

**Keywords:** personalized feedback, Web-based intervention, student alcohol consumption

## Abstract

**Background:**

Alcohol consumption in the student population continues to be cause for concern. Building on the established evidence base for traditional brief interventions, interventions using the Internet as a mode of delivery are being developed. Published evidence of replication of initial findings and ongoing development and modification of Web-based personalized feedback interventions for student alcohol use is relatively rare. The current paper reports on the replication of the initial Unitcheck feasibility trial.

**Objective:**

To evaluate the effectiveness of Unitcheck, a Web-based intervention that provides instant personalized feedback on alcohol consumption. It was hypothesized that use of Unitcheck would be associated with a reduction in alcohol consumption.

**Methods:**

A randomized control trial with two arms (control=assessment only; intervention=fully automated personalized feedback delivered using a Web-based intervention). The intervention was available week 1 through to week 15. Students at a UK university who were completing a university-wide annual student union electronic survey were invited to participate in the current study. Participants (n=1618) were stratified by sex, age group, year of study, self-reported alcohol consumption, then randomly assigned to one of the two arms, and invited to participate in the current trial. Participants were not blind to allocation. In total, n=1478 (n=723 intervention, n=755 control) participants accepted the invitation. Of these, 70% were female, the age ranged from 17-50 years old, and 88% were white/white British. Data were collected electronically via two websites: one for each treatment arm. Participants completed assessments at weeks 1, 16, and 34. Assessment included CAGE, a 7-day retrospective drinking diary, and drinks consumed per drinking occasion.

**Results:**

The regression model predicted a monitoring effect, with participants who completed assessments reducing alcohol consumption over the final week. Further reductions were predicted for those allocated to receive the intervention, and additional reductions were predicted as the number of visits to the intervention website increased.

**Conclusions:**

Unitcheck can reduce the amount of alcohol consumed, and the reduction can be sustained in the medium term (ie, 19 weeks after intervention was withdrawn). The findings suggest self-monitoring is an active ingredient to Web-based personalized feedback.

## Introduction

Alcohol consumption in the student population continues to be cause for concern [1-3]. Heavy episodic or binge drinking is prevalent in this population (eg, [4]), increasing the risk of engaging in risky, illegal, and violent behaviors [5-7]. In addition to the immediate personal and societal costs associated with alcohol misuse, heavy consumption during college and university is predictive of alcohol dependence in later life. Despite this, help-seeking behavior for alcohol use is low in the student population [8], meaning relatively few students access the traditional support services available.

Building on the established evidence base for traditional brief interventions, interventions using the Internet as a mode of delivery are being developed. Such developments have potential to aid early identification and reach their targets on a population level. Emerging evidence suggests that interventions targeted at eHealth care systems aimed at reducing harmful alcohol use that are implemented as part of a wider health care system can be cost-effective [9]. There is evidence that Internet interventions with and without therapist support can provide cost-effective behavior change with those drinking at harmful levels [10]. The potential for eHealth interventions to intervene early and engage non-help-seeking individuals means eHealth solutions for providing personalized feedback to the general population hold the potential to increase effectiveness and cost-effectiveness of public health interventions. The cost-effectiveness of this approach requires further investigation. But the ability to engage individuals in personalized feedback on a population basis combined with an ability to enable confidential access at a time convenient to the user makes electronic delivery of interventions attractive.

There is evidence that Web-based interventions that provide personalized feedback and incorporate social norms information can be effective in moderating alcohol use [11-14]. Conventional approaches to alcohol and drug health education were based upon an assumed lack of knowledge concerning the risks associated with drinking alcohol. These risk-focused campaigns are increasingly viewed as ineffectual [15]. In particular, it is acknowledged that risk-based campaigns may be dismissed by the target population due to the relatively low occurrence of risk events within the general population [16].

The social norms approach recognizes that people tend to overestimate the alcohol consumption of others and that these misperceptions predict heavier alcohol use [17,18]. There is growing evidence that interventions that include instant personalized social norms feedback can reduce alcohol consumption [19]. Recent reviews, however, have pointed to inconsistencies in reported effectiveness and efficacy. These differences can be explained by weaknesses in the methodological quality of some evaluations [19-22] and by differences in the immediacy of feedback [23]. Reviews have highlighted the need for further studies that utilize rigorous research designs [20-22] and that include longer follow-up data [21,24].

Published evidence of replication of initial findings and ongoing development and modification of Web-based personalized feedback interventions for student alcohol use is relatively rare. Exceptions include the body of work investigating e-CHUG [25,26], Unitcheck [12,27], and developments following the e-SBI pilot trial conducted by Kypri [11,28,29].

The current paper reports on the replication of the initial Unitcheck feasibility trial [27]. The feasibility randomized controlled trial (RCT) recruited 506 participants from a single UK university. After completing an online assessment, intervention participants received brief electronic personalized feedback. The intervention was available over a 12-week period, and participants could log on at any time and receive instant feedback. The trial reported a significant difference in Time 1 (week 1) to Time 2 (week 12) alcohol consumed per occasion. However, no significant difference was found for units of alcohol consumed over the previous week (1 UK unit=10 mL ethanol). As a feasibility study, the trial had a number of methodological shortcomings. No information was collected on daily alcohol intake so it was not possible to examine possible intervention effects on drinks per day over the previous week. As data were collected at only two time points (week 1 and week 12), the trial could say nothing about the short- to long-term effect of the intervention. There is a need for additional research that seeks to replicate, and understand further, initial findings and how intervention developments affect outcome. The current study sought to address these limitations and to evaluate the intervention in a larger sample.

Accordingly, the aim of the current study was to evaluate the effectiveness of Unitcheck, and the hypothesis tested was that use of Unitcheck would be associated with a reduction in alcohol consumption.

## Methods

### Setting

The study was an RCT conducted at the University of Leeds, a UK university located in the Yorkshire and Humber region of England. During the time that this study was undertaken, not all non-clinical RCTs were expected to be registered (Multimedia Appendix 2).

### Procedure and Participants

**Figure 1:**
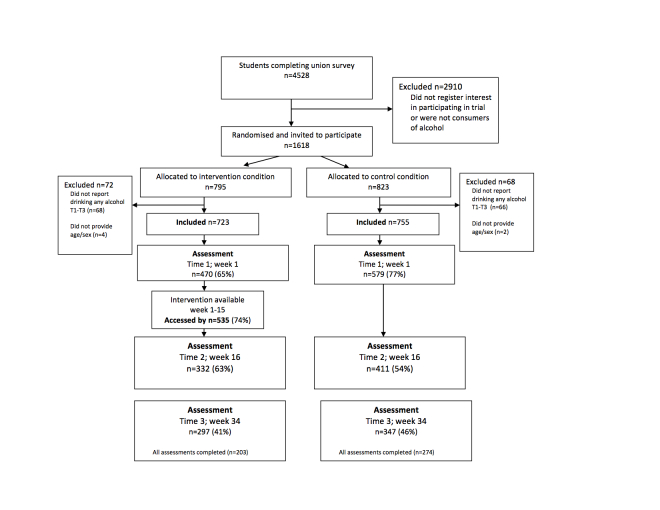
Participant flow through the trial.

In January 2007, students completing a university-wide annual student union electronic survey (n=4528) were invited to participate in a study investigating student alcohol consumption. Students who registered their interest, gave initial online consent, and provided data at baseline indicating they were a consumer of alcohol (n=1618; Time 0=T0) were invited to participate in the current study (see Figure 1). Participants were asked to complete online assessments at week 1 (Time 1=T1), week 16 (Time 2=T2), and week 34 (Time 3=T3). Those allocated to receive the intervention had access to the website from week 1 to week 15. Control participants completed all self-assessments using an online survey (created using Bristol Online Survey), and intervention participants completed T1 and T2 assessments via the Unitcheck intervention website. T3 self-assessments were completed using an online survey (created using Bristol Online Survey). Participation was anonymous. Response rates at each time point were as follows: Time 1, 65% (n=1049); Time 2, 46% (n=743); and Time 3, 40% (n=644). The intervention was accessed by 74% (n=535) of participants allocated to the intervention condition.

As an incentive to participate in the study, participants received university printer credits depending on their level of participation, with the maximum total amount (150 printer credits valued at £1.50) being given to individuals in the intervention condition who completed T1 (week 1), T2, and T3 assessments and also visited the site during week 7. The maximum total amount available to control participants was valued at £1.25.

The study was approved by Leeds East NHS Research Ethics Committee.

### Research Design

The study was an RCT with two arms: a control arm (assessment only) and an intervention arm (access to a website providing instant personalized feedback). Participants were stratified by sex, age group, year of study, self-reported weekly alcohol consumption (classified by department of health risk level) and randomly assigned (by a researcher not involved in the current study) to one of the two arms. Participants were not blind to allocation.

Data were collected electronically via two websites: one for each treatment arm. Both websites included the same questions presented in the same order. Contact with participants was by email, and at each stage participants received a standardized message inviting them to participate in the study. Each message included a direct link to the appropriate Web-based survey. Those who did not initially respond to the study were sent an email reminder once a week for up to 3 weeks. All participants were informed that they would be randomly allocated to a control (ie, assessment only) or an intervention arm. Immediately after completing the T1 assessment intervention participants received personalized feedback and social norms information. Intervention participants had access to the intervention website between T1 and T2 (15 weeks), and there were no restrictions placed on the number of visits they could make to the site. Those in the intervention arm received an additional email invitation to visit the intervention website at week 7.

### Sample Size

The distribution of alcohol units consumed over the last week is skewed; transformed data is closer to being normally distributed. This adds distributional validity to our modeling. From previous work we ascertained that the average natural logarithm of the number of units of alcohol consumed over the last week plus 1 for students is approximately 1.3 with a standard deviation of 0.58 and, hence, a variance of 0.34. Sample size determination is based on a matched-pairs *t* test. A change in natural logarithm plus 1 over the intervention period will therefore have a variance of less than 0.68 (2 times 0.345, or the variance of first measure plus the variance of the second). We have taken it to be equal to 0.49 (ie, 0.7^2^).

The difference in the change between two treatment arms might be tested with a *t* test where the relevant standard deviation is 0.7. A suitable difference in change in the natural logarithm of the number of units consumed over the last week plus 1 was taken as 0.2, so that we sought a standardized difference of 0.29. For a significance level of alpha equal to 0.05 and 90% power, a sample size of 258 participants per treatment arm was required. To allow for attrition, we aimed to recruit at least 688 participants in total.

A change of 0.2 in log(units+1) corresponds to a change in units of around 4-5 units at the average level of drinking of 21 units per week.

### Assessments

The CAGE is an assessment that was widely used as a screening tool for alcohol use disorders [30,31]. It consists of four items: (1) have you ever thought about Cutting down on your drinking, (2) do you ever get Annoyed at criticism of your drinking, (3) do you ever feel Guilty about your drinking, and (4) do you ever have a drink in the morning (an Eye-opener). Scoring positively on two or more of the items indicates problem drinking. The CAGE has previously been used within college populations [32] and has good internal consistency (alpha values between 0.52 and 0.90; [33]).

Participants were asked to report the typical number of alcoholic drinks they usually consume per drinking occasion (collected T1-T3) and how many alcoholic drinks they consumed over the last week (collected T0-T3) using a 7-day retrospective drinking diary. This method is recommended for use within samples that consume alcohol regularly [34]. The diary included a list of common alcoholic beverages and for each day of the last week/per average occasion asked participants to indicate how many of each drink they had consumed over the relevant time period. The number of alcoholic drinks consumed was then converted into UK units of alcohol consumed (1 unit=10 mL ethanol). As a result of completing the drinking diary, the number of days of alcohol consumption per week was also recorded. Weekly unit consumption was subsequently categorized according to UK government guidelines [35], namely, within recommended weekly guidelines (female 0-14 units, male 0-21 units), hazardous weekly consumption (female 15-35 units, male 22-50 units), and harmful weekly consumption (female >35 units, male >50 units). For the purposes of providing feedback, those drinking at hazardous levels were further split into two categories (female 14-21 units, male 22-28 units and female 22-35 units, male 29-50 units).

In order to assess risk behavior, participants were asked if, in the last 12 months they had experienced the following: injury to self accidentally, deliberate self-harm, injury caused by others who have been drinking, damage to property while drinking, and sexual intercourse when they ordinarily would not.

### Intervention

Unitcheck provides immediate, fully automated, personalized feedback on alcohol consumption and social norms information. This feedback was available every time participants visited the website and completed the online assessment. Unitcheck was available to those in the intervention arm from weeks 1 to week 15. (An example of feedback offered and how feedback content differed from Bewick [27] can be found in Multimedia Appendix 1). The online personalized feedback consisted of three main sections:

(1) Feedback on level of alcohol consumption: Participants were presented with statements indicating the number of alcohol units they consumed per week and the associated level of health risk. Statements were standardized for each risk level (within recommended, hazardous, harmful), and gave advice about whether personal alcohol consumption should be reduced or maintained within the current sensible levels. The number of alcohol-free days was indicated, alongside information stating that it is advisable to have at least two per week. Statements related to binge drinking behavior (ie, drinking at least twice the recommended daily limit in one session) were also presented.

(2) Social norms information: Personalized statements were presented that indicated to participants the percentage of students who report drinking less alcohol than them. This was calculated relative to the risk level generated in section 1 of the feedback, and the frequency of students within each risk level was taken from data collected as part of an earlier university wide survey investigating aspects of student life in Leeds [36]. Information was also provided about the negative effects of alcohol intake reported by students who consume similar amounts of alcohol (ie, who are within the same risk category).

(3) Generic information: standard advice was provided on calculating units, the general health risks of high levels of consumption, and outlined sensible drinking guidelines publicized in the United Kingdom. Tips for sensible drinking and the contact details of both local and national support services were also presented.

### Data Analysis

Previous research has suggested differential attrition according to treatment arm, and some trials have observed relatively high rates of attrition. These trial characteristics render the traditional repeated measures MANCOVA problematic, specifically liable to dropout bias. Therefore an analysis of the primary outcome data was planned that could accommodate these characteristics [37]. In order to assess the effectiveness of the intervention, the primary outcome variable was units consumed over the past week. The data were modeled using a multilevel longitudinal regression model with time points clustered within students. That is, regression of the natural logarithm of the number of units plus 1 regressed upon male sex, assigned to intervention, age, total CAGE score, number of visits to the intervention website, and risk-taking behavior. The model was fitted on a log scale, and we took the exponential to present results on the original scale of units. It was possible that any observed effect of intervention could have been artificially produced by differential dropout rates, eg, heavier drinkers may have been less likely to complete assessments. Therefore, a logistic regression model was fitted to predict who would not complete the study. Age, units consumed the previous week at T0, sex, and treatment arm were included in the regression model. Specifically, multiple imputation was not undertaken since it depends upon the assumption that data are Missing At Random (MAR)—considered not to be likely in this situation.

Descriptive means and standard deviations were calculated for the CAGE total score, units of alcohol consumed per week and per occasion at T1, T2, and T3. Regression analysis was carried using Stata version 11.0, and descriptive statistics were carried out using SPSS v15. The data for units per week and per occasion were positively skewed, and the data were transformed before analysis was conducted. The means and standard deviations reported in the text and tables are based on untransformed data.

## Results

Of the 1618 students randomly allocated, 1124 (69%) were female. Participants’ age ranged from 17-50 years (mean years 20.8, SD 3.2). The majority of participants (87%) were undergraduate students, and 84% were white/white-British, based on self-reported choice from among several categories of ethnicity. The majority of the sample were UK (85%), full-time (97%) students. All 1618 students were invited to participate in the current trial. The current analysis reports on the n=1478 participants who accepted the invitation. The corresponding figures for the demographics of those who provided demographic data and are included in the current analysis are: n=1036 (70% of 1478) female, age range 17-50 years old, n=1279 (88% of 1453) white/white British, n=1282 (88% of 1459) UK student, n=1438 (99% of 1459) full-time students. Table 1 summarizes these demographics by treatment arm allocation.

### Alcohol Consumption and Behavior

Of 1478 participants, 50% (n=737) reported consuming alcohol within UK government recommended weekly guidelines, 38% (n=556) at hazardous levels, and 13% (n=185) at harmful levels. Students reported consuming on average 12.7 units per occasion (SD 10.8) and 21.1 units over the last week (SD 20.9). See Table 2 for consumption by treatment arm allocation.

**Table 1:** Demographics of participants at baseline by treatment arm allocation (number of participants who provided demographic data is provided underneath demographic variable; percentages calculated as a percentage out of participants who provided variable data).

	Control n=755	Intervention n=723	Total n=1478
Female, n (%) n=1478	543 (71.9)	493 (68.2)	1036 (70.1)
Age, mean (SD) n=1454	20.8 (3.50)	20.8 (3.09)	20.8 (3.30)
Undergraduate, n (%) n=1459	666 (88.2)	626 (86.6)	1292 (88.6)
Full-time, n (%) n=1459	733 (98.5)	705 (97.5)	1438 (98.6)
UK student, n (%) n=1459	664 (89.2)	618 (85.5)	1282 (87.9)
White/white British, n (%) n=1453	658 (88.7)	621 (87.3)	1279 (88.0)

**Table 2:** Units per occasion, per previous week, and CAGE total score by treatment arm.

Consumption		Time 0		Time 1		Time 2			Time 3	
		n	M (SD)	n	M (SD)	n	M (SD)	n		M (SD)
**Units consumed over the previous week** ^a^										
	Control	755	21.7 (20.9)	544	18.0 (18.5)	380	16.3 (17.5)	321		17.1 (16.5)
	Intervention	723	20.6 (20.9)	457	16.2 (16.2)	325	13.7 (15.0)	281		16.5 (18.4)
**Units consumed on average drinking occasion** ^a^										
	Control	741	12.7 (9.75)	544	10.64 (7.26)	380	10.70 (6.67)	321		9.50 (5.49)
	Intervention	711	12.7 (11.8)	457	9.82 (7.13)	325	8.36 (6.21)	281		8.44 (4.87)
**CAGE total score**										
	Control			539	1.91 (1.19)	377	1.88 (1.23)	316		1.78 (1.22)
	Intervention			436	1.87(1.23)	295	1.751 (1.28)	272		1.75 (1.27)

^a^This table presents untransformed data while analysis was carried out on transformed data.

Regarding negative consequences and risk-taking behavior as a result of drinking within the past year: 34% (n=333) had injured themselves accidentally, 27% (n=248) had been injured as a result of someone else’s drinking, 22% (n=195) had sexual intercourse when they ordinarily would not, 10% (n=93) had damaged property, and 3% (n=30) had caused harm to self.

### Effectiveness of the Personalized Feedback and Social Norms Intervention

The variables included in the longitudinal regression model were assessment of units consumed over the last week at T1, T2, and T3; treatment arm allocation; sex; age (in years); and number of visits to intervention website. Total CAGE score, units consumed on an average drinking occasion, and reported risk taking were excluded from the final model as they did not add significantly to the model fit. The longitudinal regression model showed a significant effect of completing assessment (without intervention) on change across time with the assessment effect being greatest for those who completed T3 assessment. The model also predicted an additional effect of being assigned to intervention arm, being female, being older, and repeat visits to the intervention website.

Table 3 provides details of the regression coefficients fitted in the longitudinal model. In addition an intercept term of 3.58 corresponded to the outcome, log (1 +units consumed). It should be noted that the model identifies a lack of balance between control and intervention group at T0; the intervention group had fewer heavy drinkers. This imbalance is seen despite the stratification by unit consumption detailed in the method and despite raw observed mean values of last week consumption being similar between arms (see Discussion for further comment; see Table 2). The model yielded an overall *R* ^2^ value of 0.05 and an interclass correlation coefficient of .24, indicating that there was significant variation between participants and over time. The transformation makes the model hard to interpret directly, and so we have calculated examples in Table 4. For example, the model predicted that a typical 21-year-old female allocated to control who completed T1 assessment would, at week 34, drink 13.33 units per week while the corresponding figure for males was 19.89 units. As can be seen in Table 4, when students completed T3 assessment, consumption decreased to 12.43 for females and 18.54 for males. When assigned to the intervention arm, there was an additional effect with the model predicting that at week 34 females in the intervention condition would drink 9.49 units per week while males would drink 14.15 units. There was an additional effect of multiple visits to the intervention website. The model predicted females who visited the site three times would drink 5.87 units per week while males would drink 8.76 units. Despite the variation in individual drinking patterns across time, the data included enough observations to see an effect of the intervention.

Regarding adherence, a typical participant completed between two and three of the four assessments (mean assessments 2.6). The logistic regression model showed that the risk of dropping out after baseline was increased by being assigned to the intervention and drinking more at baseline; neither sex, age, nor total CAGE score added significantly to the model once these variables were taken into account. After completing T1 assessment, there was no clear pattern concerning dropout—attrition appeared to be random and not predicted by any of the covariates recorded.

**Table 3:** Table of coefficients for longitudinal regression model: log (1+units consumed over the last week) regression on assessment completed, condition allocation, sex, age, and number of visits to website by restricted maximum likelihood.

Covariate	Coefficient	95% CI	*P* value
Complete assessment T1	-.15	-0.25 to -0.06	.001
Complete assessment T2	-.36	-0.47 to -0.25	<.001
Complete assessment T3	-.24	-0.35 to -0.13	<.001
Allocated to receive feedback	-.27	-0.41 to -0.13	<.001
Male	.40	0.32 to 0.48	<.001
Age	-.04	-0.05 to -0.03	<.001
Number of visits to feedback website	-.16	-.21 to -0.11	<.001
Constant	3.58	3.32 to 3.84	<.001

**Table 4:** Prediction of units consumed over the last week at each time point (longitudinal regression model).

	Female 21 years old	Male 21 years old								
	Allocated to control	Allocated to intervention					Allocated to control	Allocated to intervention				
# of visits to intervention		0	1	2	3	4		0	1	2	3	4
Completed assessment at T0	15.49	11.82					23.10	17.64				
Completed assessment at T1	13.33	10.18					19.89	15.18				
Completed assessment at T2	10.80	8.25	7.03	5.99	5.10	4.35	16.12	12.30	10.49	8.94	7.61	6.49
Completed assessment at T3	12.43	9.49	8.08	6.89	5.87	5.00	18.54	14.15	12.06	10.28	8.76	7.46

## Discussion

### Principal Findings

This study aimed to evaluate the effectiveness of Unitcheck. The model predicted a monitoring effect, with participants who completed assessments reducing alcohol consumption over the last week. Further reductions were predicted for those allocated to receive the intervention, and additional reductions were predicted as the number of visits to the intervention website increased. The model therefore supported the hypothesis that Unitcheck, a Web-based social norms intervention, can reduce the amount of alcohol consumed over the last week. The model did not predict a reduction of units consumed on an average occasion. The results also suggest that the reduction can be sustained in the medium-term (ie, 19 weeks after access to the intervention was closed).

The previous feasibility trial reported significant reductions in units consumed per occasion but not in units consumed over the last week [27]. In this replication study, assessment of units consumed over the last week was carried out by providing participants with a list of common alcoholic beverages and asking them to indicate how many they had consumed over the last 7 days. In the current trial, the assessment was altered; participants were provided with a list of common alcohol beverages and were asked to indicate how many they had consumed on each day over the last 7 days (ie, 7-day recall). The current sample reported higher levels of consumption when compared to the feasibility sample. It is unclear whether this difference is due to differences in recording or actual behavior.

The current study findings are consistent with our multisite trial [12] that observed an effect of assessment across time on units consumed in the previous week; an additional effect of being assigned to receive the intervention was also predicted. The current study predicted a monitoring effect, and the multisite study results supports this finding with the greatest reductions being observed among participants who were monitored (ie, completed at least 2 of the 5 assessments). In both studies, there was an additional effect of being allocated to the intervention arm.

It is a strength of the current study that participants reported a range of levels of consumption (from within sensible guidelines to hazardous drinking). Unitcheck was designed as a public health intervention that could be delivered across the student population. In contrast, previous studies have reported a large proportion of low-level consumers [28], limiting the potential to see any significant decrease in consumption.

Since, after T1, dropping out is not related to previous drinking behavior, the changes in drinking are not due to completers being the lighter drinkers; this is a further strength of the study. Prior to completing T1, the risk of dropping out was increased by being assigned to the intervention and drinking more at baseline. This is consistent with previous research report of higher levels of attrition among heavier consumers of alcohol [27,38]. This suggests further work is needed to consistently engage students who are currently consuming alcohol at potentially problematic levels. In addition, it is necessary that we understand the processes by which participants choose to engage with research investigating Web-based interventions and, ultimately, how to encourage increased levels of engagement with interventions.

A common method used to investigate the influence of dropout from longitudinal studies is multiple imputation. Multiple imputation is dependent on the assumption that data are MAR. In the current study, we consider MAR unlikely; therefore, multiple imputation was not used in the analysis.

### Limitations

This RCT included a medium-term postintervention follow-up. This, combined with the relatively large numbers of participants recruited and retained (compared with previous studies in this area [22,27]), means it makes a distinctive contribution to the evidence base. However, a number of limitations need to be considered when interpreting the results. First, the intervention group had fewer heavy drinkers. This does not necessarily detract from the findings reported but is an issue for concern. The attempt to stratify by four confounders was too ambitious. As a consequence, the stratification by alcohol units was too crude and the imbalance occurred. Second, the study design randomized individuals after registering interest but before providing full baseline assessment. This meant that 71% of those randomized accepted the invitation to participate and provided T1 assessment. Third, although 74% of intervention participants accessed the intervention, the proportion who engaged with follow-up assessments was lower (with 43% of intervention participants completing all assessments; 47% of control participants). High dropout is a concern since it might explain the findings rather than the monitoring or intervention. For example, if heavier drinkers drop out, then the average level of drinking of those retained will decline over time. To explore this, we investigated models for dropout. There was evidence of an association between heavier drinking and dropout after T0 but not beyond that time. We note also that at T3, the average level of drinking increases rather than decreases; this is inconsistent with the “alternative” but consistent with effects of monitoring and intervention wearing off over time. Fourth, while there was a 34-week follow-up assessment, these results say little about the longer-term impact of the intervention. The longevity of electronic brief interventions is still uncertain, but the current results suggest that repeated access to such interventions might help maintain behavior change. Fifth, participants were not blind to their condition as participants were aware of whether or not they received feedback. Control participants were aware that at the end of the study they would gain access to personalized feedback. Sixth, there were two small differences in the treatment of the intervention and control groups (intervention participants could receive up to £0.25 more than control participants; intervention participants received an extra email contact reminding them to visit the website).

### Conclusions

These results lend further support to the efficacy and potential effectiveness of using Web-based interventions to reduce alcohol consumption among the student population. The findings add weight to the suggestion that one active ingredient to Web-based personalized feedback is the self-monitoring support they afford to individuals. By adding a postintervention follow-up, this study supports the idea that behavior change instigated as a result of engaging with Web-based interventions can be sustained, at least in the short- to medium-term. Future research should seek to investigate the generalizability of these findings to other sections of the general population. In addition, further work is needed to understand the mechanisms of engagement and behavior change, in the hope of further enhancing the impact of brief Web-based interventions.

## Multimedia Appendix 1

Original feedback with details of how feedback was altered between the feasibility study (Bewick et al, 2008) and the current study.

## Multimedia Appendix 2

CONSORT-EHEALTH checklist V1.6.2 [39].

## Abbreviations

CAGE: (1) have you ever thought about Cutting down on your drinking, (2) do you ever get Annoyed at criticism of your drinking, (3) do you ever feel Guilty about your drinking, and (4) do you ever have a drink in the morning (an Eye-opener)
MANCOVA: multivariate analysis of covariance
MAR: missing at random
RCT: randomized controlled trial
