# Supplementary material for: The Effectiveness of a Web-Based Personalized Feedback and Social Norms Alcohol Intervention on United Kingdom University Students: Randomized Controlled Trial
Source: J Med Internet Res. 2013 Jul 24;15(7):e137. doi: 10.2196/jmir.2581 (PMC3742391; doi:10.2196/jmir.2581)
Supplement: Supplementary file 1 [file jmir_v15i7e137_app1.pdf]

The current effectiveness study used an amended version of the Bewick et al (2008) study website. Changes made to the original eUNICAL (the earlier version of Unitcheck) website are detailed in the blue text boxes. Further changes were made before the implementation of the Bewick et al (2010) multi-site study.

Altered so information was given for 'last week' but followed up by a statement concerning whether 'last week' was typical (and if not typical a statement of risk associated with 'typical' week).

Alteration so feedback now reads:

- Over the last week you drunk XX units
- On an average occasion you typically drink XX units

[illegible] you have given. After viewing your feedback make sure you tell us [illegible]utton below.

How much am I drinking?
You have indicated that you drink 45 units per week

Is my health at risk ?

Your weekly alcohol consumption puts your health at MODERATE RISK. In order to minimise health risks you need to cut down your alcohol intake. If you feel you need help changing your drinking behaviour then please contact one of the help services below.

How much is a unit?

- A pint of ordinary strength lager (e.g. Carling Black Label, Fosters) = 2 Units
- A pint of strong lager (e.g. Stella Artois, Kronen bourg 1664) = 3 units
- A pint of bitter (John Smith's, Boddingtons) = 2 units
- A pint of ordinary strength cider (e.g. Dry Blacthorn, Strongbow) = 2 units
- A 175ml glass of red or white wine = around 2 units
- A pub measure of spirits = 1 unit
- An alcopop (e.g. Smirnoff Ice, Bacardi Breezer, WKD, Reef) = around 1.5

Responses you have given to other questions has also indicated that you have some concerns regarding your drinking behaviour (for example you may have indicated that you have felt guilt about your drinking and/or have thought about cutting down on your drinking). If you would like to discuss these concerns with anyone, please see the list of contact numbers below.

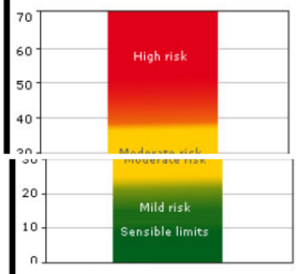

Risk categories depending on units of alcohol consumption for males

Alcohol free days

You've indicated that you have at least 2 alcohol free days a week. Making sure you always have these will minimise health risks.

What is sensible drinking?

Women 0-13 units per week, Men 0-20 units per week

You should have at least 2 ALCOHOL FREE DAYS a week

You should break up your drinking with non- alcoholic drinks

You should drink no more than 1 unit of alcohol an hour

Am I a binge drinker?

The amount you are drinking per occasion classifies you as a binge drinker. Binge drinking puts risks on your health. To minimise these risks you should cut down the amount you drink on each occasion.

How could my health be at risk?

Too much alcohol .

• can irritate the stomach, leading to sickness and nausea. • causes dehydration and insomnia • can affect nearly every organ in your body if drinking occurs over a prolonged period. • can cause liver damage and high blood pressure, which can contribute to heart disease. • greatly increases calorie intake, perhaps leading to weight gain, • cause loss of libido and fertility in men and women. • increases the risk of depression and emotional disorders.

[illegible]t is binge drinking?

[illegible]king SIX or [illegible]e units in a row on [illegible]gle occasion.

Binge drinker feedback extended to enable feedback based on units on average occasion AND responses on AUDIT that suggest has previously consumed at this level

How much do other students drink?

At least 75% of Leeds University undergraduate students drink less than you. Only 20% of students drink a similar amount of alcohol to you (within MODERATE RISK). Of Leeds University undergraduate students who also drink within MODERATE RISK.

- Almost 90% feel that their drinking has a negative effect on their finances.
- Approximately 50% of students feel that it has a negative effect on their studies and/or their physical health.

If you also have any concerns about the negative effects of alcohol on aspects of your life then please contact one of the help services below.

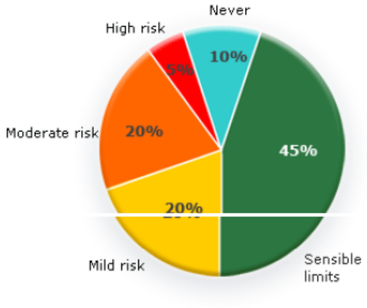

Students reported level of alcohol consumption

Disclaimer : The information on this site is not intended as medical advice and should only be used as a guide to understanding your alcohol use and the potential health issues involved with it. It is not a substitute for professional care so if you have or suspect you may have an alcohol related problem, you should seek professional attention.

The e-UNICAL team would like to know how you have found the survey, website and the feedback that has been provided. Please click on 'continue' at the bottom of the page and let us know your comments.

If you feel you need help or advice on any concerns raised from this feedback then please contact one of the following help services.

Nightline (Information Line) 0113 380 1380
Nightline (Listening Line) 0113 380 1381
Student Counselling Centre 0113 343 4107
Drinkline 0800 917 8282

The Samaritans 08457 90 90 90
NHS Direct 0845 4647
Your local GP

submit and continue Print Logout
